# Study protocol: a hybrid effectiveness-implementation trial of Moral Reconation Therapy in the US Veterans Health Administration

**DOI:** 10.1186/s12913-018-2967-3

**Published:** 2018-03-07

**Authors:** Daniel M. Blonigen, Michael A. Cucciare, Christine Timko, Jennifer S. Smith, Autumn Harnish, Lakiesha Kemp, Joel Rosenthal, David Smelson

**Affiliations:** 1 Center for Innovation to Implementation, VA Palo Alto Health Care System, Palo Alto, CA USA; 2 Palo Alto University, Palo Alto, CA USA; 3 Department of Psychiatry and Behavioral Sciences, Stanford University School of Medicine, Palo Alto, CA USA; 4 Center for Mental Healthcare and Outcomes Research, Central Arkansas Veterans Affairs Healthcare System, North Little Rock, AR USA; 5 Department of Psychiatry, University of Arkansas for Medical Sciences, Little Rock, AR USA; 6 Center for Health Care Organization and Implementation Research, Bedford VA Medical Center, Bedford, MA USA; 7 Veterans Justice Programs, Veterans Health Administration, Washington DC, USA; 8 University of Massachusetts Medical School, Worcester, MA USA

**Keywords:** Moral Reconation therapy, Justice-involved veterans, Randomized controlled trial, Veterans health administration, Hybrid trial, Effectiveness, Implementation

## Abstract

**Background:**

Moral Reconation Therapy (MRT) is a cognitive-behavioral intervention aimed at reducing risk for criminal recidivism by restructuring antisocial attitudes and cognitions (i.e., “criminogenic thinking”). MRT has empirical support for reducing risk for criminal recidivism among civilian offenders. Recently, a version of MRT was developed for military veterans; however, no randomized controlled trials (RCT) have been conducted with the veteran-specific protocol, and the effectiveness and implementation potential of MRT outside of correctional settings has not been established.

**Methods:**

Using a Hybrid Type 1 RCT design, this study will test the effectiveness of MRT to reduce risk for criminal recidivism and improve health-related outcomes among justice-involved veterans entering mental health residential treatment at three US Veterans Health Administration (VHA) Medical Centers. Upon admission to the treatment program, justice-involved veterans will complete a baseline assessment, be randomized to usual care (UC) or UC + MRT, and be followed 6 and 12 months post-baseline. A process evaluation will also be conducted to identify barriers and facilitators to implementation of MRT in residential treatment.

**Discussion:**

The primary aim of this study is to evaluate the effectiveness of MRT with justice-involved veterans. If MRT proves effective in this trial, the findings can provide large healthcare systems that serve veterans with an evidence-based intervention for addressing criminogenic thinking among justice-involved adults, as well as guidance on how to facilitate future implementation of MRT in non-correctional settings.

**Trial registration:**

This trial is funded by the VA Health Services Research & Development Program (IIR 14–081) and is registered with ClinicalTrials.gov (ID: NCT02524171).

## Background

Among criminal offenders, two-thirds will recidivate (i.e., be rearrested, reconvicted, or reincarcerated for a new crime or violation of their parole or probation) within 3 years from their release [1]. Criminal recidivism is also common among veterans of the US military (“justice-involved veterans”), who comprise 8% of the incarcerated population in the US (approximately 181,500 individuals). For example, the majority of incarcerated veterans in US jails and prisons have at least one prior episode of incarceration, and 43% have four or more prior lifetime arrests [2]. Therefore, there is a critical need to develop and test interventions that may reduce criminal recidivism in this population.

According to the Risk-Need-Responsivity model of offender rehabilitation, antisocial cognitions and attitudes – referred to as *criminogenic thinking* – are the risk factors most strongly associated with criminal recidivism [3, 4]. Criminogenic thinking has also been linked to offending among US military veterans [5–9]. Cognitive-behavioral interventions that are designed to restructure antisocial cognitions and behaviors represent best practices for reducing criminal recidivism [10–13]. However, the availability of these interventions for justice-involved veterans following release from correctional settings is less than the availability of services to address other risk factors for recidivism identified in the Risk-Need-Responsivity model – e.g., substance abuse [14].

Moral Reconation Therapy (MRT) is a cognitive-behavioral intervention that aims to reduce criminogenic thinking [15] and has the strongest empirical support for reducing criminal recidivism among civilian offenders [8]. MRT is a manualized intervention that uses an open-enrollment, group format and includes a patient workbook. The MRT curriculum consists of structured exercises and homework assignments that aim to modify the antisocial cognitions and behaviors of participants in order to move them through 12 steps of moral development. The term “conation” has been employed to describe the conscious process of decision-making and purposeful behavior. The term “moral reconation” was chosen for this treatment approach because the underlying goal is to change conscious decision-making to higher levels of moral reasoning [16].

Multiple meta-analyses in civilian populations support the efficacy of MRT to reduce criminal recidivism [10, 11, 17–20]. For example, in a review of 65 studies, MRT was found to reduce the 12-month recidivism rate by 50% [20]. More recently, a meta-analysis of 33 published studies of MRT found that the rate of recidivism among MRT participants is reduced by one-third compared to the rate of those who do not receive MRT and that the treatment effect size is greater in randomized (vs. non-randomized) trials. Further, a structured evidence review from the US Department of Veterans Affairs examined the evidence base for MRT in civilian samples [8]. Six studies were identified as being rigorous enough to provide interpretable evidence about the impact of MRT and all demonstrated a significant reduction in reoffending in the MRT group [21–26].

Aside from reductions in criminal recidivism, MRT has theoretical and empirical associations with improvement in several health-related outcomes such as substance use [21, 27], mental health [28–30], housing [31], and employment [21]. The theoretical and empirical links between MRT and these health-related outcomes are important, given that these health-related outcomes are risk factors for future recidivism, and improvements in these outcomes have been shown to reduce recidivism. For example, according to the Risk-Need-Responsivity model [32], substance use and employment problems are robust predictors of criminal recidivism and are key domains measured by valid indices of recidivism risk [33].

Despite its evidence base in civilian samples, no randomized controlled trials (RCT) of MRT have been conducted with justice-involved veterans. This is an important research gap as justice-involved veterans differ from justice-involved civilians on sociodemographics (justice-involved veterans tend to be older, more educated, and more likely to be married) [34, 35], offense characteristics (justice-involved veterans are more likely to have committed violent offenses, particularly intimate partner violence) [34–36], mental and physical health problems (justice-involved veterans have more service-related traumas and traumatic brain injuries) [37, 38], and interpersonal problems (a strong connection to military culture in veterans can increase feelings of estrangement from social networks upon return to civilian life) [39]. Given these differences, prior research on MRT with civilian populations may not be generalizable. In response to this gap in the literature, a version of MRT specific to justice-involved veterans was developed in 2013 [16]; however, its efficacy with this group of offenders has yet to be examined.

Another issue is that MRT was originally developed for use within correctional settings in which treatment can be mandated and rewards and sanctions for treatment engagement or non-engagement, respectively, can be applied. Consequently, knowledge regarding the effectiveness and implementation potential of MRT in non-correctional settings is limited. Understanding the implementation of MRT in non-correctional settings is important given the rise of diversion programs and specialty courts in the US over the past decade [40]. Moreover, mental health services are increasingly called upon to treat criminal offenders and reduce their risk for criminal recidivism. However, there is a lack of data on the effectiveness and implementation potential of MRT within mental health treatment programs. To fill this gap in the literature, the study described in this protocol paper aims to evaluate the effectiveness and implementation potential of MRT for veterans in mental health residential treatment programs within the US Veterans Health Administration (VHA) – a large, integrated healthcare system which provides mental health and psychosocial services to a substantial number of justice-involved veterans [41–43].

## Method/design

### Study design

This study will use a Hybrid Type 1 design [44], which is a mixed-methods design that will allow us to test the effectiveness of MRT in an RCT across three VHA medical centers, as well as gather qualitative data on MRT’s implementation potential in a process evaluation. The overarching objective of the current study is to evaluate MRT as an intervention to reduce risk for criminal recidivism and improve health-related outcomes among justice-involved veterans in VHA mental health residential treatment programs. At each study site, veterans who (a) are entering a mental health residential treatment program, and (b) had been arrested and charged and/or released from incarceration in the past 5 years will be recruited for participation and randomly assigned to one of two conditions: usual care (UC) or UC + two MRT group meetings per week for 12 weeks.

The specific aims of this study are to determine whether adding MRT to usual care in mental health residential treatment reduces overall risk for criminal recidivism (Aim 1, Hypothesis 1a); improves health-related outcomes that are risk factors for recidivism (i.e., substance use, mental health, housing, and employment problems) (Aim 1, Hypothesis 1b); and whether the effects of MRT on reduced recidivism risk and better health-related outcomes is mediated in part by greater likelihood of completing the residential treatment program and utilizing substance use disorder/mental health continuing care services (Aim 1; Hypothesis 1c). Patients will be followed 6 and 12 months post-baseline. The process evaluation will entail qualitative interviews with mental health treatment program providers and patients (Aim 2). The goal of this aim is to inform the future implementation of MRT in VHA by identifying barriers and facilitators to implementation in residential treatment programs.

In addition to these aims, the study design will also allow us to explore other mediators and moderators of MRT’s effects. For example, improvements in interpersonal functioning and reductions in affiliations with antisocial/substance-using peers are key mechanisms of MRT. Therefore, we will examine if these variables mediate links between receipt of MRT and better outcomes during the follow-up period. Finally, given that (a) psychopathic personality traits have been found to moderate recidivism among justice-involved adults [45], and (b) patients in this trial will have varied histories of criminal justice involvement, we will explore whether level of psychopathy and criminal history at baseline moderate MRT’s direct effects on outcomes.

### Setting

To increase the generalizability of findings, participants will be drawn from mental health residential treatment programs at three large VHA Medical Centers on the West Coast, Midwest, and East Coast of the US. These residential programs are analogous to the settings in which MRT was originally developed (i.e., drug therapeutic communities in prison) and serve a high proportion of justice-involved veterans who have extensive histories of criminal justice involvement [46].

### Participants

Veteran patients who (a) are entering a mental health residential treatment program at one of the participating sites, (b) had been arrested and charged and/or released from incarceration in the 5 years prior to their current admission to residential treatment, and (c) are conversant in English will be eligible for participation and recruited into the RCT portion of the study. Exclusion criteria are being too cognitively impaired to understand the informed consent process and other study procedures, and pregnancy. Power analyses were calculated to determine the sample size needed to obtain a small-to-medium effect size (i.e., *f*^2^ = .07) with 80% power. To account for having patients at three sites, the analyses were based on six cells (two conditions per site). Based on an alpha of .05 (two-tailed), the inclusion of three moderator variables and three interaction terms, and an expected attrition rate of 20% at the follow-up assessments, a total of 365 patients will need to be recruited into the study. The sample will be stratified by site such that 122 patients (with rounding) will be recruited from each of the three VHA medical centers, with 61 patients entering each condition at each site.

For the process evaluation (Aim 2), we will interview six staff members from the mental health residential treatment programs at each site who were involved in the delivery and/or implementation of MRT. We will also conduct interviews with 12 veteran participants from each site who were randomized to the MRT condition: one-half will be drawn from those who remained engaged in MRT during their stay in the residential program, and the other half from those who dropped out of MRT prior to being discharged from the residential program. Staff interviews will take place after all patient participants have completed the intervention phase of the RCT. Patient interviews will take place after participants have either completed or dropped out of the intervention.

### Recruitment for RCT

Veteran patients entering mental health residential treatment programs at participating sites will be screened by treatment program staff in consecutive order during the admissions process to determine their eligibility for the RCT. Treatment program staff members will also verify that the veteran is not pregnant and is conversant in English. Treatment program staff will then ask eligible patients for permission to be contacted about the study and will provide study personnel with the names and contact information of eligible patients.

Eligible patients will be contacted by study personnel, who will explain the purpose of the study, what study participation would involve, and the risks and benefits of participation. Patients will be informed that they will be assigned either to “usual care” or two additional one-hour long group sessions per week in the residential treatment program. Regardless of their group assignment, patients will also be informed that they will be asked to complete an in-person baseline assessment and two follow-up assessments (in-person or via phone) 6 and 12 months later. Patients who express interest in participating will be scheduled for the baseline assessment. At the beginning of that assessment, patients will be screened for cognitive impairment using the Montreal Cognitive Assessment’s section on Orientation [47]. Patients who are not able to correctly answer the Orientation (date, location) items will be deemed too cognitively impaired to understand the study’s procedures, participate meaningfully in MRT, and respond to interviews, and therefore will be ineligible. For those who are eligible, written informed consent will be obtained, and the baseline assessment will be administered.

### Randomization

At the beginning of the study before participants are recruited, a randomization schedule will be established for each site using www.randomizer.org. Randomization will occur in fixed block sizes of six to assure a roughly equal balance of participants in the two conditions in case the study does not fully accrue. The schedule concealment will be maintained by the principal investigator at each study site. At each site, after creation of the randomization spreadsheets, sealed envelopes will be labeled with a unique study ID number and include the condition assignment based on the randomization spreadsheet. Following completion of the baseline assessment, the research assistant will open the envelope for that unique study participant, notify the participant of his/her group assignment, and schedule the first MRT session for those randomized to that condition. Patients randomized to the UC condition will be asked to not seek out MRT during the 12-month study period. To reduce contamination, patients in the UC condition will not be allowed to attend MRT sessions, and the MRT group facilitators will remind the members at the end of each session not to discuss the group content outside of the session. In the event the study team learns that MRT-related information was shared with UC participants, the records of those UC participants will be flagged, and contamination-adjusted intention-to-treat analyses will be calculated (see Data analysis section) [48].

### Usual care

All patients, regardless of condition, will receive usual care in the mental health residential treatment program at one of the three sites. Mental health residential treatment programs serve veterans dealing with homelessness, substance abuse, and/or mental health issues. These programs aim to improve veterans’ health and facilitate community reintegration by providing care in a structured residential environment. The residential programs are comparable across sites in terms of program structure and services delivered (patients are involved in therapeutic activities 7 h per day, 5 days per week), clinical approaches (individual and group-based, cognitive-behavioral programming for substance use and mental health problems) and staffing (psychiatrists, psychologists, social workers, nurses, addiction therapists, vocational therapists, homelessness coordinators). The participants’ health care providers in the residential treatment programs (not the study team) will be providing usual care. No standard treatment will be withheld.

### Intervention

Patients assigned to the MRT condition will receive usual care in the residential treatment program plus 2 h of MRT weekly. Over the course of 12 weeks, they will attend 24 group sessions (one-hour sessions, twice per week). The overall length of the intervention (12 weeks) corresponds to the national average of bed days of care for veterans in mental health residential treatment (3 months) and the minimum intended length of stay for the target residential programs in this study. Regardless of patients’ length of stay, continuing care services are available at the residential programs at each site, and those who have not yet completed the full MRT protocol when they discharge from the residential programs will be encouraged to continue with the intervention as outpatients.

The MRT curriculum consists of short assignments, grounded in cognitive-behavioral techniques, which move participants through 12 steps that aim to restructure antisocial cognitions and behaviors. To progress through the steps, patients complete homework between group meetings and then present their homework to group members in the following week’s meeting for feedback. Participants move through steps at a rate of approximately 1–2 sessions and group sessions can incorporate new members at any time. With open enrollment, patients are presenting on different steps, which is advantageous because those who have progressed farther are able to share their insights to newer patients who are at lower steps. To facilitate patients’ engagement in MRT, small reinforcements (coins and certificates) will be offered for completion of key steps in the MRT curriculum (i.e., Steps 3, 7, and 12).

To assess ongoing fidelity to the MRT condition, at each site, one group session per month will be observed in-person by study research staff and evaluated against a fidelity checklist that has been used in prior research with MRT. The completed fidelity checklists for each site will then be reviewed each month by the site PIs and discussed on a monthly treatment fidelity call with the developers of MRT who will provide corrective feedback and consultation to the MRT group facilitators. A subset of group sessions will also be audio- or video-recorded (with participant consent) to permit evaluation of inter-rater reliability of the checklist coding.

### Data collection

*RCT.* For the RCT, research assistants will collect data from patients at three time-points: baseline, and 6 and 12 months post-baseline. The baseline assessment will be conducted in-person approximately 1 week after the patient is admitted to the residential treatment program. The 6- and 12-month follow-up assessments will be conducted either in-person or via telephone. We will use an intent-to-treat design and follow all patients who are randomized to either the UC or MRT condition. An overview of the variables that will be measured across the study time-points and the specific measures or data sources used to index these variables are shown in Table 1. Collectively, the measures represent validated tools that have been commonly used in prior studies of criminal justice and/or substance-using populations. With exception of the Triarchic Psychopathy Measure, all measures will be administered at all time-points.

**Table 1:** Variables and measures/data sources for the randomized controlled trial

Variables	Measures/data source
Recidivism risk (overall)	Psychological Inventory of Criminal Thinking Styles
Substance use	ASI Alcohol and Drug modules Timeline Follow-back Interview (alcohol and drug use calendars)
Mental health	ASI Psychiatric Status module (Global) Patient Health Questionnaire-9 (Major Depression symptoms) PTSD Checklist-DSM-5 version (PTSD symptoms)
Housing	VHA’s Homeless Operations Management and Evaluation System
Employment	ASI Employment/Support module
Completion of the mental health residential treatment program	VHA administrative records
Substance use disorder/mental health continuing care utilization	VHA administrative records Alcoholics Anonymous Interview
Interpersonal functioning	ASI Family/Social module Inventory of Interpersonal Problems
Antisocial/substance-using peer affiliations	Measure of Criminal Attitudes and Associates Life Stressors and Social Resources Inventory
Psychopathic personality traits	Triarchic Psychopathy Measure *
Criminal history/recidivism	ASI Legal Status module

*Notes.* * = only administered at baseline (all other measures will be administered at all time-points – baseline, 6 months, and 12 months). ASI = Addiction Severity Index. VHA = Veterans Health Administration

Outcome selection was guided by Risk-Need-Responsivity framework [3, 4], which describes the most robust risk factors for criminal recidivism, as well as Fontaine’s work on homelessness and recidivism [49, 50]. The primary outcome of *recidivism risk* will be measured with the Psychological Inventory of Criminal Thinking Styles [51], a self-report measure used to assess antisocial cognitions and behaviors (i.e., “criminogenic thinking”). Items on this measure are summed to create a General Criminal Thinking score, which has been validated as an overall index of recidivism risk [52, 53]. Although reductions in criminal recidivism is the ultimate goal of MRT, given the non-correctional setting, as well as the fact that patients in this trial will have varied histories of criminal justice involvement, it is anticipated that the base rate of criminal recidivism will be low during the 12-month follow-up period. Therefore, we elected to focus on *recidivism risk* as the primary outcome and to index this outcome via a measure of criminogenic thinking, given it is the strongest dynamic risk factor for recidivism [3, 4].

Secondary outcomes include health-related outcomes of *substance use* (via the Alcohol and Drug modules of the Addiction Severity Index [ASI] [54], and Timeline Follow-back interviews for alcohol and drug use) [55], *mental health* (via the Psychiatric Status section of the ASI to index global mental health, the Patient Health Questionnaire-9 to index symptoms of major depression [56], and the PTSD Checklist, DSM-5 version to index symptoms of PTSD [57]), *housing* (via items from the VHA’s Homeless Operations Management and Evaluation System) [58], and *employment* (via the Employment/Support section of the ASI).

In order to test whether the effects of MRT on reduced recidivism risk and better health-related outcomes are mediated in part by greater likelihood of completing the mental health residential treatment program and utilizing substance use disorder/mental health continuing care services, we will also collect data on service utilization (via patients VHA administrative records) and mutual-help group attendance (via the Alcoholics Anonymous Interview [59]). In order to conduct exploratory mediation analyses, we will measure *interpersonal functioning* (via the Family/Social functioning module of the ASI and the Inventory of Interpersonal Problems [60]) and *antisocial/substance-using peer affiliations* (via select items from the Measures of Criminal Attitudes and Associates [61] and the Life Stressors and Social Resources Inventory [62]) to test whether changes in these measures mediate links between receipt of MRT and better outcomes during the follow-up period. To conduct exploratory moderation analyses, we will collect information on *psychopathic personality traits* (via the Triarchic Psychopathy Measure [63] and *criminal history* (via the Legal Status module of the ASI) to test whether scores on these indices at baseline moderate MRT’s direct effects on outcomes. The ASI Legal Status module will also be used to measure *criminal recidivism* during the follow-up period.

### Process evaluation

The RE-AIM (Reach, Effectiveness, Adoption, Implementation, and Maintenance) Planning Tool [64] will be used to guide the content of the semi-structured phone interviews with mental health residential treatment program providers and patients at each site. The RE-AIM framework [65] highlights five domains to evaluate an intervention’s potential for implementation and widespread impact: *Reach* (how to reach the target population with the intervention); *Effectiveness* (how to know the intervention is effective); *Adoption* (organizational support to implement an intervention); *Implementation* (fidelity and consistency of an intervention’s delivery); and *Maintenance* (sustainability of an intervention in the long-term). The RE-AIM Planning Tool will be modified to ask questions of providers and patients within these domains regarding key issues that should be considered when planning to implement MRT in mental health residential treatment across VHA.

### Data analysis

#### RCT

Intent-to-treat analyses will be conducted. The primary and secondary outcomes will be measured at all three time-points. Therefore, we will use generalized linear mixed model (GLMM) regressions to compare the UC and MRT conditions on these outcomes. These models take into account that the repeated measures across time points are clustered within each individual by treating time as a single repeated factor. GLMMs also have the advantages of being able to use all available data (under the assumption that data are missing at random) and being able to accommodate different conditional distributions for outcome variables (e.g., dichotomous; continuous; Poisson-distributed). We will also examine these outcomes as a function of MRT dose (i.e., number of group sessions attended; number of steps completed). To test mediation, we will conduct GLMM regressions following the approach of Mackinnon and Fairchild [66], which will correspond to a causal sequence among condition, the hypothesized mediator, and the outcomes, and control for covariates (e.g., baseline scores on the outcome). To test moderation, we will explore whether at baseline various indices of criminal history (i.e., incarcerated or not in the past 12 months; number of months since charged with an offense or released from incarceration; and extent of criminal history) and psychopathic personality traits moderate the direct effects of condition on the primary and secondary outcomes. Finally, to address the possibility of contamination, if we learn that MRT-related information was shared with UC participants, the records of those UC participants will be flagged, and contamination-adjusted intention-to-treat analyses will be calculated [48] (i.e., the effect of treatment assignment on outcomes is adjusted by the percentage of participants assigned to the UC condition who may have received the treatment).

#### Process evaluation

The de-identified audio-files of the staff and patient interviews will be transcribed. The interview transcripts will then be will be analyzed using template analysis to identify common themes in textual data [67]. This approach allows for identification of codes a priori, as well as modification of codes and addition of new codes based on a reading and interpretation of the data. This process will be used to identify themes related to barriers and facilitators to MRT implementation at the system, provider, and patient levels. These barrier and facilitator themes will be summarized and categorized into tables, which will include potential solutions and potential-to-leverage columns that contain evidence-based implementation tools to be considered for more widespread usage of MRT.

## Ethical considerations

This study was reviewed and approved by the VA’s Central IRB (Study #15–04) and registered with ClinicalTrials.gov (ID: NCT02524171). Prospective participants will be told that they may withdraw from the study at any time and refrain from answering specific questions, and that all information will be confidential and used only for the purposes of the research study. Prospective participants will also be told that any withdrawal from the study on their part or on the part of the investigators will not affect the standard care that the participant is receiving in the residential treatment program or any other healthcare from the VHA to which the participant is entitled.

## Discussion

The primary aim of this hybrid trial is to evaluate the efficacy of MRT with justice-involved veterans using the new veteran-specific protocol of this intervention [16]. Another key contribution of this trial is the opportunity to learn about factors that may either hinder or facilitate implementation of MRT in a non-correctional setting. As noted above, MRT was originally developed for use within correctional settings. Thus, knowledge of the implementation potential of MRT in large, integrated healthcare systems such as the VHA is limited, and unique challenges to (or opportunities for) implementation of MRT in this setting may emerge in this trial. This issue is timely, given that recent policy efforts in the US that are focused on diversion rather than incarceration (e.g., specialty courts) have shifted the burden of treating justice-involved adults and reducing their risk for recidivism from correctional services to behavioral health services in the community [40].

Recent work by Blonigen and colleagues [68] highlights a number of potential challenges to implementation of MRT in the VHA. One such challenge that is relevant to the current trial is patient engagement, given the time commitment involved in MRT. Specifically, the MRT curricula and homework assignments that patients are required to complete between group sessions can be time intensive, and completion of all 12 MRT steps requires at least 24 sessions, on average. Consequently, engagement may be challenging for non-incarcerated populations. This may be due to the fact that for those who are not incarcerated or are not mandated to attend MRT by the criminal justice system, there are no consequences for not engaging in the intervention. In addition, those who are not incarcerated may have other demands on their time such as employment and/or other treatment requirements placed on them by the criminal justice system or the residential program in which MRT has been implemented. We will address this issue by offering reinforcements (coins and certificates) for completion of key steps in the MRT curriculum (e.g., Steps 3, 7, and 12). For patients who are still under supervision of the criminal justice system, such incentives are often highly valued as it allows them to demonstrate to court-related decision makers that they are complying with treatment [69, 70]. In addition, there is evidence to suggest that completion of the first seven of the 12 steps in MRT is associated with long-term reductions in criminal recidivism [71, 72]. This suggests that a lower dose than the 24 sessions that are planned for patients randomized to MRT in this trial may still yield benefits on the primary and secondary outcomes. We estimate 12 sessions as the minimal dose of MRT for this study. To maximize the clinical usefulness of the data, our analytic plan includes an examination of the effect of condition on outcomes as a function of (a) number of MRT group sessions attended, and (b) number of MRT steps completed. Finally, the potential challenges of patient engagement in MRT in non-correctional settings and how to properly incentivize participation (or sanction non-participation) will be a key focus of the process evaluation, which will entail interviews with patients who did or did not remain engaged in MRT during their residential stay.

This hybrid trial represents the first attempt to systematically evaluate the effectiveness of MRT with justice-involved veterans and the implementation potential of this intervention in a large, integrated healthcare setting. The potential impact of this work is underscored by the extensive criminal histories of veterans treated in mental health residential treatment programs in VHA [46] and the dearth of empirically-supported treatments in this healthcare system that directly address criminogenic thinking [14] – the strongest dynamic risk factor for criminal recidivism [3, 4]. By testing and implementing MRT in residential treatment programs, where many patients have extensive criminal histories, integrated healthcare systems such as the VHA can better understand how to reduce risk for criminal recidivism among patients with criminal justice system involvement and in turn improve the long-term health and well-being of this vulnerable population.

## Abbreviations

ASI: Addiction Severity Index
GLMM: Generalized Linear Mixed Models
MRT: Moral Reconation Therapy
RCT: Randomized Controlled Trial
RE-AIM: Reach, Effectiveness, Adoption, Implementation, and Maintenance
UC: Usual Care
VHA: Veterans Health Administration
